# Genome Holography: Deciphering Function-Form Motifs from Gene Expression Data

**DOI:** 10.1371/journal.pone.0002708

**Published:** 2008-07-16

**Authors:** Asaf Madi, Yonatan Friedman, Dalit Roth, Tamar Regev, Sharron Bransburg-Zabary, Eshel Ben Jacob

**Affiliations:** 1 School of Physics and Astronomy, Tel Aviv University, Tel Aviv, Israel; 2 Faculty of Medicine, Tel Aviv University, Tel Aviv, Israel; 3 Computational and Systems Biology, Massachusetts Institute of Technology (MIT), Boston, Massachusetts, United States of America; 4 The Center for Theoretical and Biological Physics, University of California San Diego, La Jolla, California, United States of America; Tel Aviv University, Israel

## Abstract

**Background:**

DNA chips allow simultaneous measurements of genome-wide response of thousands of genes, i.e. system level monitoring of the gene-network activity. Advanced analysis methods have been developed to extract meaningful information from the vast amount of raw gene-expression data obtained from the microarray measurements. These methods usually aimed to distinguish between groups of subjects (e.g., cancer patients vs. healthy subjects) or identifying marker genes that help to distinguish between those groups. We assumed that motifs related to the internal structure of operons and gene-networks regulation are also embedded in microarray and can be deciphered by using proper analysis.

**Methodology/Principal Findings:**

The analysis presented here is based on investigating the gene-gene correlations. We analyze a database of gene expression of Bacillus subtilis exposed to sub-lethal levels of 37 different antibiotics. Using unsupervised analysis (dendrogram) of the matrix of normalized gene-gene correlations, we identified the operons as they form distinct clusters of genes in the sorted correlation matrix. Applying dimension-reduction algorithm (Principal Component Analysis, PCA) to the matrices of normalized correlations reveals functional motifs. The genes are placed in a reduced 3-dimensional space of the three leading PCA eigen-vectors according to their corresponding eigen-values. We found that the organization of the genes in the reduced PCA space recovers motifs of the operon internal structure, such as the order of the genes along the genome, gene separation by non-coding segments, and translational start and end regions. In addition to the intra-operon structure, it is also possible to predict inter-operon relationships, operons sharing functional regulation factors, and more. In particular, we demonstrate the above in the context of the competence and sporulation pathways.

**Conclusions/Significance:**

We demonstrated that by analyzing gene-gene correlation from gene-expression data it is possible to identify operons and to predict unknown internal structure of operons and gene-networks regulation.

## Introduction

Microarray technology produces vast amounts of raw data regarding the system-level response of the genome. Advanced analysis methods were devised to extract meaningful information from gene expression data, most of which focused on distinguishing between groups of subjects (e.g., cancer patients vs. healthy subjects) or identifying the most relevant genes that help to distinguish between those groups-marker genes that exhibit distinct up- or down-regulation.

Current DNA-expression data analysis methodologies can be divided into supervised approaches, which aim to determine genes that fit a predetermined pattern; and unsupervised approaches, which aim to characterize the components without a priori assumptions. Supervised methods are usually used to find individual genes, like in the nearest neighbor approach [1], and/or multiple genes, like in decision trees [2], neural networks [3], and support vector machines [3], [4]. Unsupervised methods are usually based on cluster analysis [5]–[10]. Several algorithmic techniques were previously used in clustering gene expression data, including hierarchical clustering [11], self-organizing maps [12], K-means [13], simulated annealing [14], and graph theoretic approaches such as HCS [15], CAST [16], CLICK [17] and bi-clique identification algorithm [18]. The study presented here was aimed to extract information about the functional (activity) relations between genes, to elicit functional sub-groups of genes and to reveal function-form motifs in an unsupervised way.

The guiding idea was that such information is embedded in the similarities (here we use Pearson correlations but other methods of similarity can also be used [19], [20]) between the expression profiles of different genes in response to different growth conditions and/or different stages of growth. Therefore, we devised an approach that is based on the analysis of gene-gene correlation matrices rather than analyzing the matrices of gene expression levels. In this regard our approach differs from other unsupervised clustering techniques-it is aim to reveal hidden properties of the network investigated and not just similar properties.

We present the new approach by analyzing a database of gene expression for a specific example in which *Bacillus subtilis* were exposed to sub-lethal levels of antibiotics [21]. The gene-expression levels were monitored in response to 37 different kinds of antibiotics and at 3 time points after the exposure. We also note that, though we focus in this work on analyzing the matrices of correlations between genes (the gene correlation matrices), important information can also be extracted, in principle, by analyzing the matrices of correlations between the responses to the different antibiotics, as we will show elsewhere.

The first step in the analysis is to evaluate, from the gene-expression data, the corresponding matrices of gene correlations. Here we employed the widely-used Pearson correlation method [22] that normalizes the correlations according to the standard deviations of the expression profile of each gene. The correlation matrices are then investigated using the functional holography (FH) method (discussed in detail in the next section and in Appendix S1). Second, in order to capture system level motifs, the FH method includes collective normalization of the correlations (according to the correlations of each gene with all the others). Third, the matrices of normalized correlations are then analyzed using dimension reduction algorithms (here we use the Principal Component Analysis algorithm–PCA [23]) to extract the most relevant information. Next, to reveal functional motifs–functional relations between genes–the genes are projected on a reduced 3-dimentinal space whose axes are the three leading principal components (PCs) of the PCA. We note that projection on a lower dimension space of PCs is a common practice in investigations using clustering approaches [24]. Last, a new element in the FH analysis is that apart from the projection on a three dimensional (3-D) space, we also draw lines between pairs of genes in the reduced space, color-coded according to the values of the correlations between the two genes. Doing so enables to retain relevant information embedded in the higher dimensions that can be lost in the dimension reduction. For example a link between genes that belong to distinct clusters in the 3-D space indicate functional connectivity in the higher dimensions. We also show that that the method enables to reveal inhibitory relations between genes that are reflected in negative correlation links.

As was mentioned above, for crucial evaluation of the new approach, we decided to apply it for gene-expression data of *B. subtilis*. The reason was that these bacteria have relatively complex genome, for which the complete DNA sequence is available, accumulated knowledge about the gene functions, exists, and the organization of genes into operons is known [25]–[30].

We tested the ability of the approach to identify, from the gene-expression data, the organization of genes into operons. These functional units in the genome are composed of one or more genes co-transcribed into one polycistronic mRNA–a single mRNA molecule that codes for more than one protein. The basic structure of an operon includes a promoter, an operator and a terminator; however, some operons (like the examples we show here) can also have quite complex internal functional organization [31]. At the macroscopic scale, operons are organized as a network connected by regulators that control, as many other biological networks, joint biological functions and pathways.

Recent advancement of experimental methods induced a rapid increase in the available detailed information about the various genes clustered in the operon system. However, knowledge is still lacking about the functional principles that govern the relationship between function and internal structure of operons as well as inter-operon regulation. We demonstrate that our analysis method can extract such information from gene-expression data.

We show that, when projecting the genes of each operon onto the 3-dimentional space according to their correlations with the other genes, they tend to form distinct clusters. Furthermore, the order of the genes within the clusters corresponds to the known form motifs of the operon (for operons whose internal structure is known) such as gene sequential order along the genome, gene separation by non-coding segments, and translational start and end regions. For operons whose structure is partially known, the analysis can elicit additional intra-operon form motifs from the measurements of the gene-responses. This approach is also capable of exposing inter-operon relationships, like operons with functional similarity, operons that share functional regulation factors, etc. In particular, we demonstrate the detection of activators and inhibitors for the competence and sporulation pathways.

## Methods

### Gene-Expression Data

**Figure 1:**
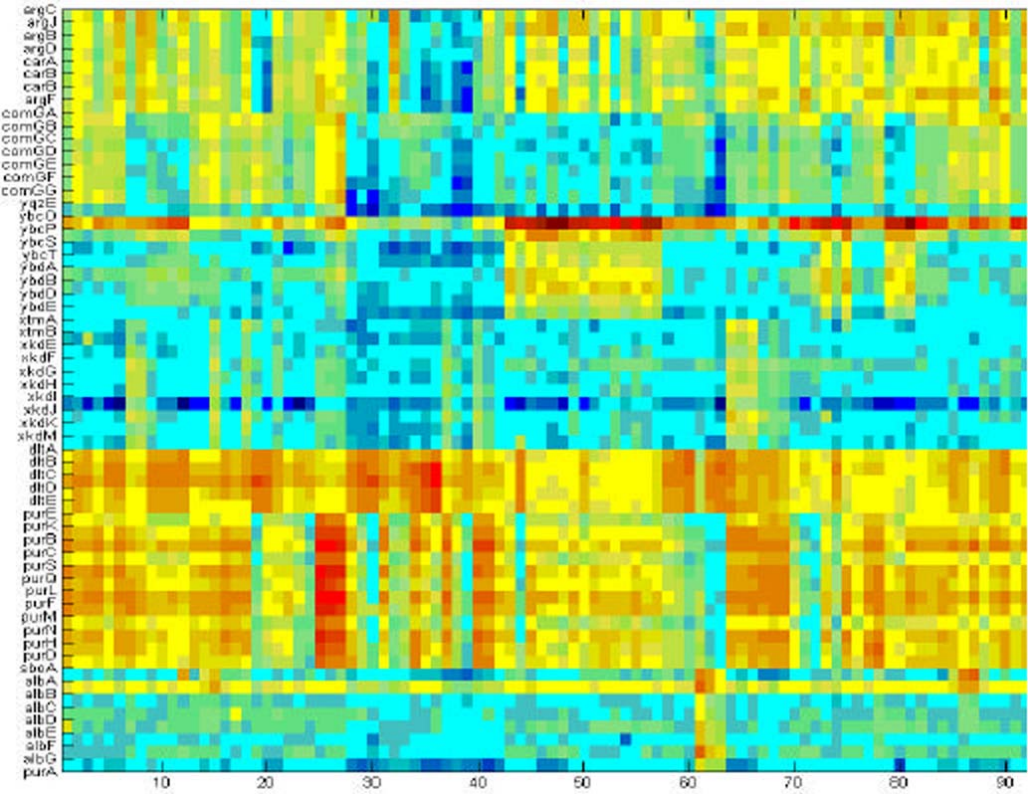
Gene expression data matrix. The expression matrix shows part of the data set [21]. This part includes the response signal of selected 59 genes to the 37 different antibiotics at 3 time points (10, 40 and 80 minutes). The matrix is organized so that each row corresponds to a specific gene. The genes [29] are sorted according to the operons they belong to. Columns 1–3 show the response to the first antibiotic at the three time points, columns 4–6 the response to the second antibiotics at the 3 time points, and so on. The particular 59 genes whose responses are shown here were selected since they belong to well known and functionally important operons.

Gene Expression data was downloaded from GPC-biotech's website: http://www.gpc-biotech.com/. The expression database constructed by Hutter *et al*. [21] presents a survey of gene expression profiling for in vivo analysis of the mechanisms of actions (MoAs) of antibacterial compounds. The database contained the transcriptional response of 4204 genes of *Bacillus subtilis* 168 following treatment with 37 antibacterial agents at three time points. The antibacterial agents were divided into six groups according to the cellular functions they affect including: cell wall biosynthesis, DNA topology, fatty acid biosynthesis, folate biosynthesis, protein biosynthesis and membrane-active compounds and ionophores. The bacteria were treated with sub-lethal (referred to in the literature also as sub-inhibitory) concentrations of each compound, i.e. at concentrations that affect the growth but do not completely halt it. RNA from each sample of treated bacteria was purified and labeled, according to the research protocol, and hybridized on a genome array generated by the research group. A list of *B. subtilis* operons and their genes was extracted from the DBTBS: database of transcriptional regulation *in B. subtilis* (http://dbtbs.hgc.jp/ [32]) The *B. subtilis* gene signals under the various antibiotics conditions are organized in a matrix–each row is the vector of gene response signals to the different antibiotics taken at the 3 time points, as illustrated in Figure 1 and Table 1.

**Table 1:** A description of the operons and their genes.

Operon Name	1-argC	2-comGA	3-ybcO	4-xtmA	5-dltA	6-purE	7-sboA
**Genes**	argC	comGA	ybcO	xtmA	dltA	purE	sboA
	argJ	comGB	ybcP	xtmB	dltB	purK	alba
	argB	comGC	ybcS	xkdE	dltC	purB	albB
	argD	comGD	ybcT	xkdF	dltD	purC	albC
	carA	comGE	ybdA	xkdG	dltE	purS	albD
	carB	comGF	ybdB	xkdH		purQ	albE
	carB	comGG	ybdD	xkdI		purl	albF
	argF	yqzE	ybdE	xkdJ		purF	albG
				xkdK		purM	
				xkdM		purN	
						purH	
						purD	

The particular 59 genes whose responses to the 37 antibiotics are shown in Figure 1 were selected since they belong to well known and functionally important operons.

### The Functional Holography Analysis

The Functional Holography (FH) approach was introduced by Baruchi *et al.,* [33]–[35] for analysis of recorded human brain activity. The term hologram stands for “whole”—holo in Greek, plus “information” or “message”—gram in Greek. The article illustrated the ability of a method to capture hidden motifs in the complex activity of neural networks and in recorded brain activity. However, the same methodology can be applied to other biological networks. In the coming paragraphs and in Appendix S1, the main features of the FH methodology and its application to the gene network will be discussed.

**Figure 2:**
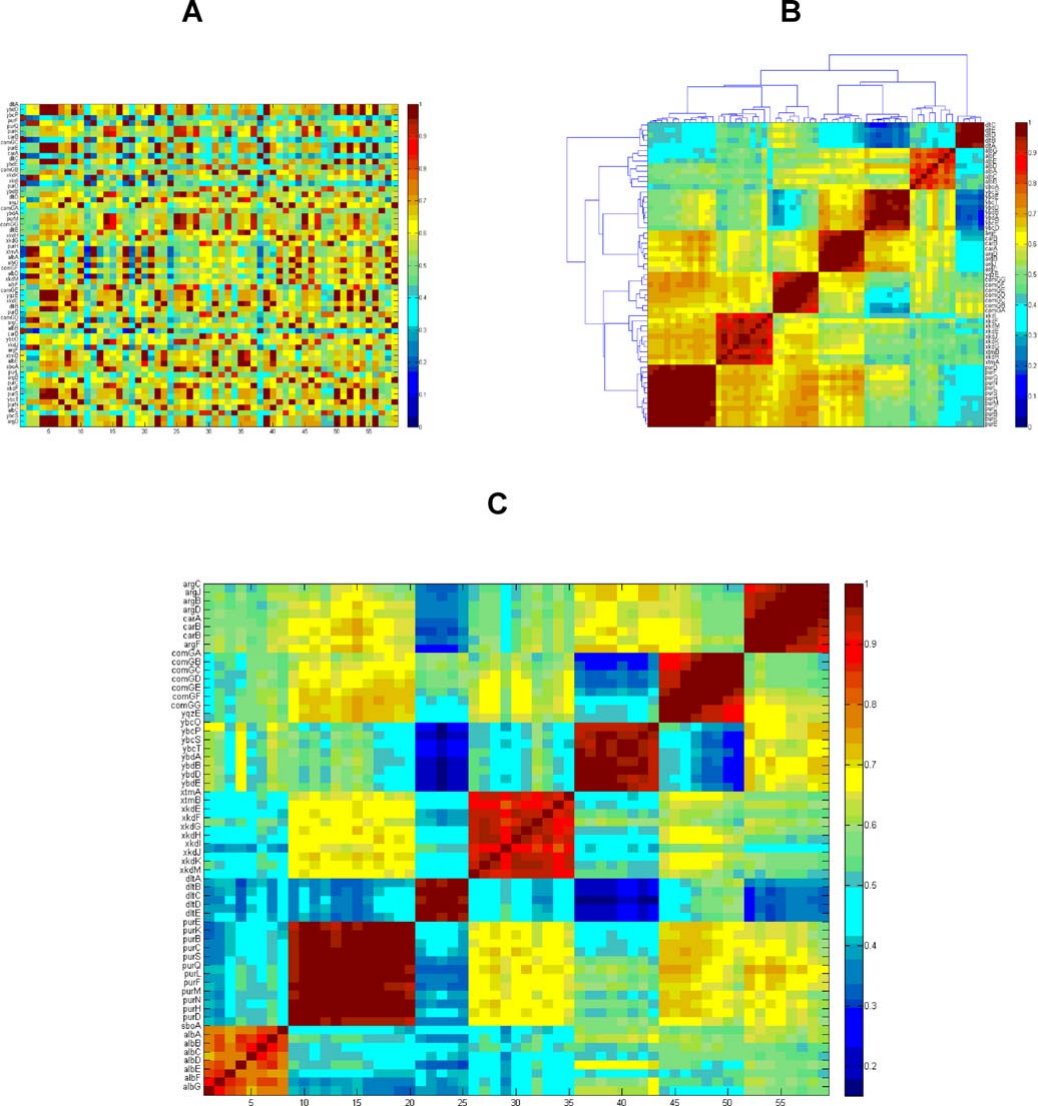
The gene correlation matrix: We show three representations (different ordering) of the gene correlation matrix for the 59 selected genes whose expression matrix is shown in figure 1. Both axes represent genes, but on the x-axis the genes are numbered to ease visualization. The Pearson correlation coefficient can be assigned values from −1 (strong anti-correlation) to +1 (strong positive correlation). Here we transformed the values from the range [−1,+1] to the range [0,1], so small values correspond to strong negative correlations. (A) The unsorted (arbitrary gene order) correlation matrix. (B) An unsupervised, sorted matrix using the dendrogram clustering algorithm [30]. (C) A supervised sorted matrix in which the genes are ordered according to the operons they belong to. In both cases, the operons form distinct clusters in the sorted matrix.

The Functional Holography analysis begins with the computation of the of gene-gene Pearson correlations matrix that corresponds to the gene expression matrix. We calculate the Pearson correlation [22] C(i,j) between the vectors X_i_(n) and X_j_(n)–the expression profiles of genes (i) and (j) for all conditions. Using this terminology, the Pearson correlation coefficient between genes (i) and (j) is given by:
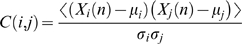
(1)The index *n* stands for the different antibiotic condition–all the antibiotics at the three time points according to the order described in Figure 1 and Table 1. The variables *μ_i_* and *σ_i_* are the mean value and the standard deviation of subject profiles (i) and (j), respectively. For illustration, the correlation matrix that corresponds to the expression matrix of the 59 selected genes (Figure 1) is shown in Figure 2 unsorted (the genes are randomly ordered), after unsupervised sorting (the correlation matrix is sorted using the dendrogram algorithm [36]), and after supervised sorting (the genes are ordered according to the operons). The Pearson correlation coefficient takes values from −1 (strong anti-correlation) to +1 (strong positive correlation). To facilitate the computations described below, we transformed the values from the range [−1,+1] to the range [0,1]. Hence we note that small values correspond to strong negative correlations.

The results show that distinct clusters are formed according to the operon classification. This implies that the correlation matrices efficiently capture the intrinsic properties of the dataset that are less transparent in the expression matrices.

#### Collective normalization

The approach also includes collective normalization or affinity transformation, discussed in details in [33]–[35]. We note that while the collective normalization helps to make the functional motifs more transparent in some cases, it is not an essential aspect of the analysis. Here, for the case of gene expression analysis, we found it best to perform the collective normalization using the meta-correlations MC(i,j). Where MC(i,j) is the Pearson correlation between the correlations of genes (i) and (j) with all other genes. Mathematically it is the correlation between rows (i) and (j) in the correlation matrix C after reordering. In the reordering process, the elements C(i,i) and C(j,j) are removed from the calculation. The correlation vector for (i) is {C(i,j), C(i,1), C(i,2),…}, and for (j) it is {C(j,i), C(j,1),C(j,2),…}. Using the meta-correlation normalization we obtain the normalized gene correlation matrix, A(i,j) given by:
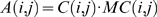
(2)The collective normalization was motivated by the idea that it can help reveal hidden collective motifs by amplification of sub-groups of strongly correlated genes and attenuation of functional relations within and between the sub-groups.

**Figure 3:**
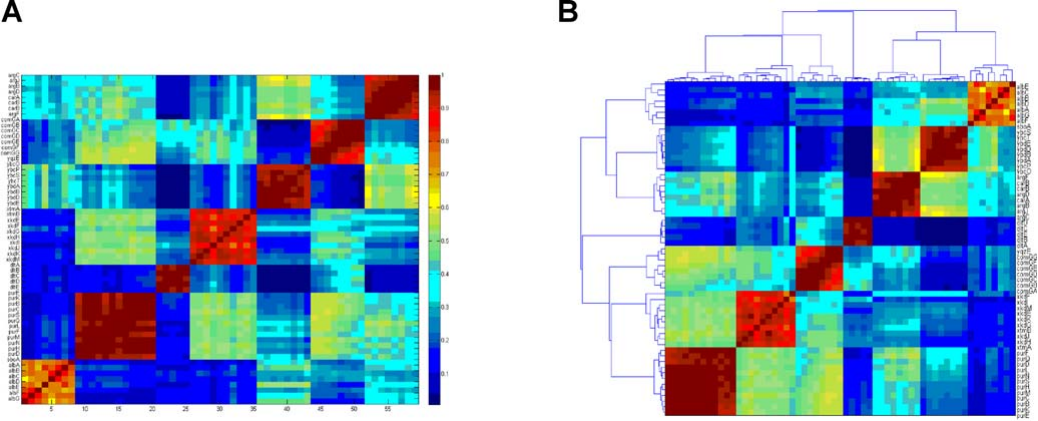
The matrix of normalized correlations. The matrix corresponds to the correlation matrix shown in Figure 2. (A) The supervised sorted matrix (according to the operons). (B) The unsupervised version, sorted by the dendrogram algorithm [30]. Note that the dendrogram algorithm sorts the operons in a different order (7,3,1,5,2,4,6), as explained in the text.

**Figure 4:**
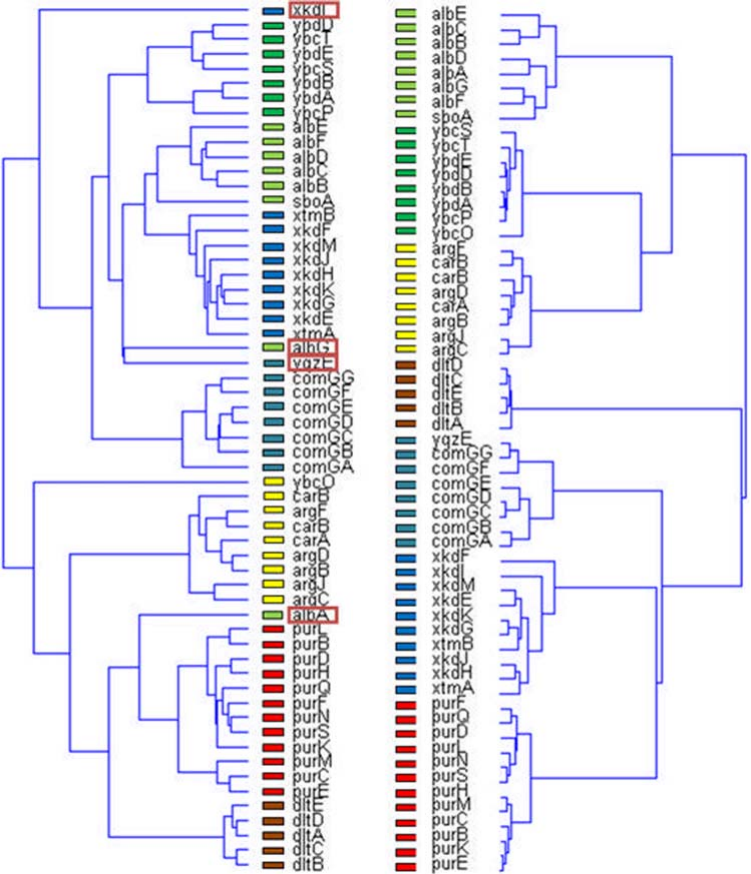
The unsupervised results of the dendrogram algorithm applied on the expression matrix (left) and on the matrix of normalized correlations (Figure 3B) (right). Genes belonging to the same operon are marked by the same color. Note that four mismatches occur only in the matrix of normalized correlations (left).

In Figure 3 we present the matrix of normalized correlations that corresponds to the correlation matrix shown in Figure 2. We present both the supervised sorted matrix and the unsupervised sorted one. We note that the separation into distinct operons is more pronounced in the matrix of normalized correlations. More specifically, the ratio R between the averaged intra- and inter-operon correlations for the correlation matrix is R = 0.204, and for the matrix of normalized correlations R = 0.357. The fraction of information (relative variance) in the first three principal components was 0.85 for the correlation matrix and 0.85 for the normalized correlation matrix, thus the normalization improves the clustering process while preserving the information from the signal. The advantages of working with the normalized correlation versus the signal can also be seen when applying unsupervised clustering such as the dendrogram algorithm (Appendix S1 and [36]) on both matrices, as shown in Figure 4. The signal matrix has four mismatches while the normalized correlation has none. For additional check, we performed the analysis was done with K-mean clustering algorithm [13] and results with similar inferiority to the signal matrix dendrogram [36] were obtained.

#### Dimension reduction

The third step in the FH analysis process aims to extract the most relevant information embedded in the normalized correlation matrix by applying a dimension reduction algorithm (Appendix S1). Here we used the Principal Component Analysis (PCA) algorithm [23] but other dimension reduction (clustering) algorithms can also be used [37], [38]. For visual representation of the functional motifs, the genes are located in a reduced 3-dimensional (3-D) PCA space whose axes are the three leading principal vectors (PC1, PC2 and PC3) of the PCA algorithm (of the corresponding covariance, matrix). Each gene (i) is located in the 3-D PCA space at a point {λ_1_(i), λ_2_(i) λ_3_(i)}, were the λ_n_(i) (n = 1,2,3) are the three decomposition eigen-values of gene (i) for the three leading principal vectors. We note that genes having high normalized correlations relative to the other pairs of genes will be closely located in the 3-D space. It is also important to note that clusters of genes imply groups of genes with functional resemblance.

#### Retrieval of lost information and the holographic network

The last step of the FH analysis process is aimed to retrieve information, embedded in higher dimensions, that might have been lost in the dimension reduction process or in the affinity transformation (i.e. non normalized correlation values) ([33]–[35] and Appendix S2). The affinity transformation can make more transparent highly correlated sub-groups, attenuate inter group correlation and at the same time retain internal functional relations within the sub-groups. Together with the PCA procedure, groups are easier to detect and analyze. For that we linked each pair of nodes by lines colored according to the original (non-normalized) correlations. In addition, we link nodes with correlations above/below a threshold, or within a range of values according to the features of interest.

The resulting manifold represents the functional relations between the genes and the connecting lines between the genes retain the information in the higher dimensions. In this sense the manifold (connectivity diagram) in the abstract 3-D space can be viewed as a “holographic network”.

## Results

### Holographic presentation of the genes

**Figure 5:**
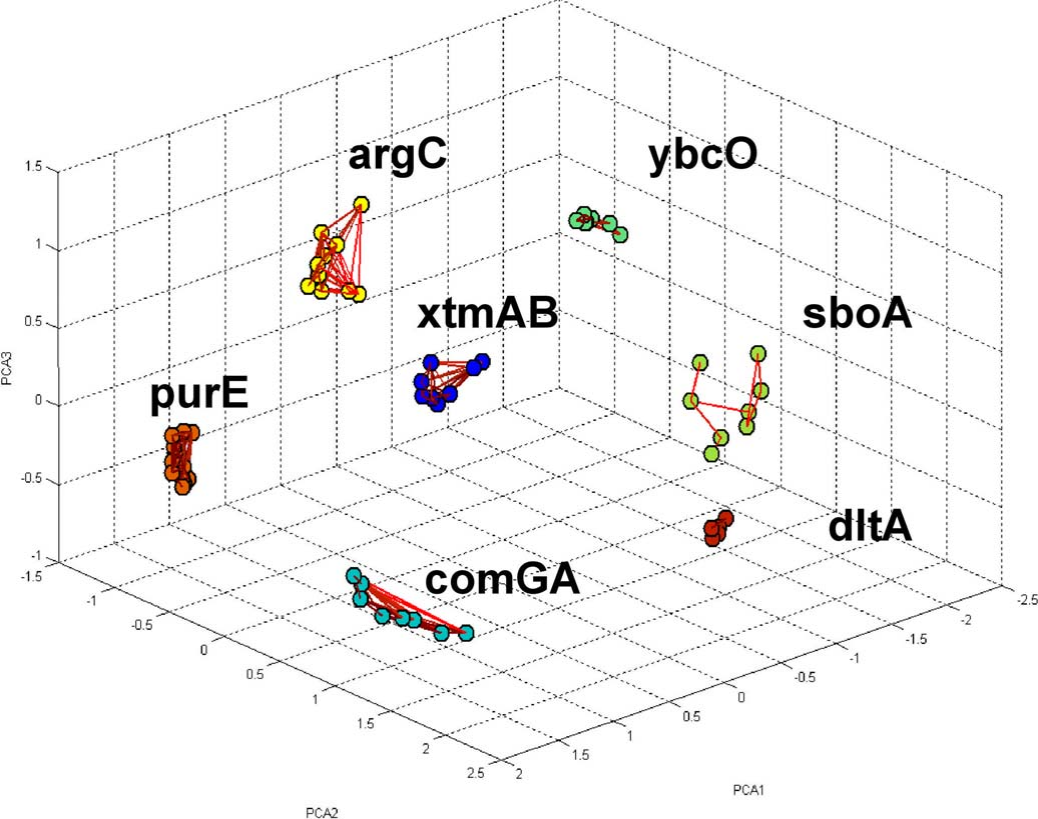
Holographic presentation of the genes in the 3-D space. The genes are located in the 3-D space whose axes are the three leading principal components calculated by applying the PCA [22] to the matrix of normalized correlations. The location of each gene is according to its three decomposition eigen-values for the three principal vectors. The links between pairs of genes are for correlations above 0.7.

In Figure 3 we showed that the normalized correlation matrix can efficiently sort the genes according to the operons they belong to. In Figure 5 we show the holographic presentation of the matrix of normalized correlations using the projection on the 3-D space as described above. Genes belonging to the same operon are marked by the same color. Pairs of genes with correlations above 0.7 are linked by lines colored according to the correlation values. As can be seen, only genes belonging to the same operon are linked, reflecting the fact that inter-operon correlations are weaker than intra-operon ones.

### Operons Holography–revealing function-form relations

We continue with holographic zooming [33]–[35] analysis of sub-groups of genes–the operons in this study. The idea is to separately perform the collective normalization on the correlation matrix of the sub-group and to calculate a new 3-D space for this specific sub-group. A clear correspondence between the genes functional relations and the known structures of the operons is revealed. The results are illustrated for two specific operons–spoVFA [39] and pyrR [40] that have non trivial internal organization.

### Holography of the spoVFA operon

**Figure 6:**
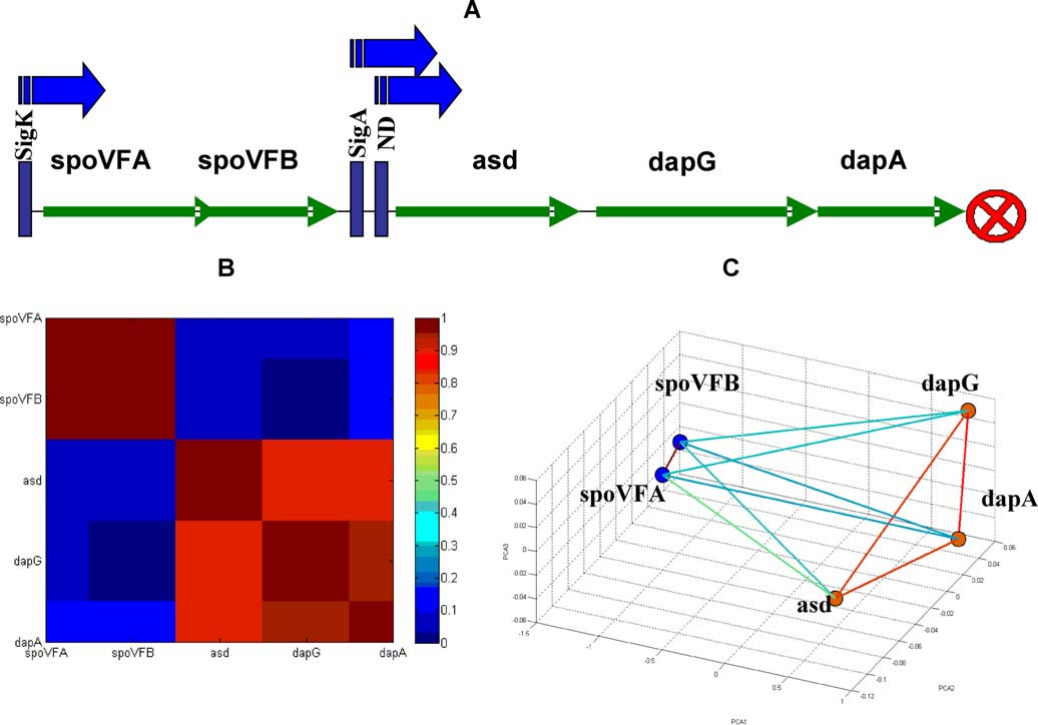
The spoVFA operon. In (A) we show schematically the known internal genomic sequential structure of this operon [39]. The operon promoters are represented by blue arrows and the terminator is represented by a red crossed circle. Binding sites of regulators sigK, sigA and ND are marked by blue rectangles. In (B), we show the ordered matrix of normalized correlations and the holographic representation of the operon is shown in (C). We note the clear correspondence between the functional relations as captured by the holographic representation and the known operon organization.

The spoVFA operon is composed of five genes that are divided into two functional sub-units (Figure 6A): 1. spoVFA and spoVFB, that are responsible for the dipicolinate synthase of subunits A and B that are involved in sporulation. 2. The asd, dapG and dapA genes that are involved in vegetative growth and in stage 5 of sporulation [41]. We can clearly observe in Figure 6B that the two sub-units form two distinct clusters in the normalized correlation matrix. In Figure 6C we see that the genes of the two sub-units are located apart and are cross-linked with weaker correlations.

### Holography of the pyrR operon

**Figure 7:**
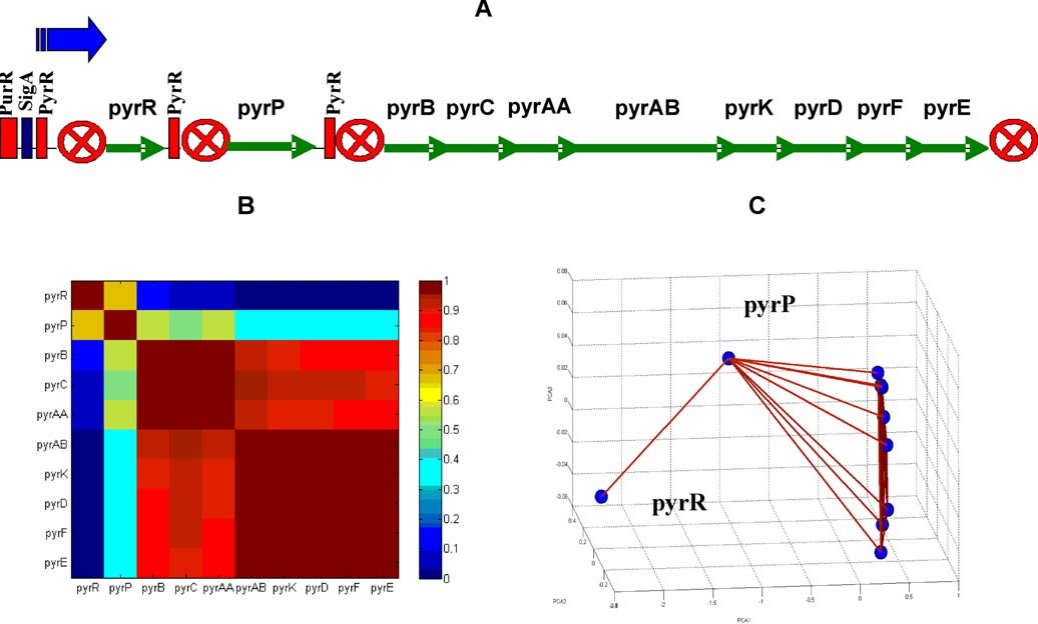
The pyrR operon. (A) Schematic internal sequential structure presentation [40]. Promoter represented by a blue arrow. Terminator represented by a marked circle. Rectangles represent regulation regions of sigA, purR and pyrR. (B) Matrix of normalized correlation. Note the low correlations between pyrR to the rest of the genes, matching its function as a negative regulator. (C) FH holographic presentation, with correlation values above 0.7 marked by lines.

The pyrR operon [40] has a more complex structure, as shown in Figure 7A. It is composed of 10 genes that are organized in three sub-units: 1. The gene pyrR that acts as self inhibitor of the operon as a whole and also acts as an inhibitor of the three sub-units. 2. The gene pyrP is located downstream from the pyrR with a terminator segment in between. 3. The third sub-unit is composed of 8 genes downstream from the pyrP with terminator and promoter segments in between. The operon is regulated by sigA and purR.

The normalized correlation matrix of the pyrR operon genes, pyrR, pyrP, pyrB, pyrC, pyrAA, pyrAB, pyrK, pyrD, pyrF and pyrE, and the corresponding holographic presentation are shown in Figure 7B,C. In Figure 7C we can clearly see that pyrR and pyrP are distinct from each other and from the 8 genes of the third sub-unit of the operon. We also note that the holographic functional organization of the operon in the 3-D space corresponds to the structural organization of the operon, as pyrR gene is linked only to the pyrP which, in turn, is linked to the rest of the genes. The genomic scheme of the operon in Figure 7A is consistent with these results, as pyrR and pyrP are separated from the rest of the genes in the operon by terminators. Furthermore, it was previously shown that pyrR is a protein that regulates the expression of genes and operons of pyrimidine nucleotide biosynthesis (pyr genes) in many bacteria and specifically in *B. subtilis*. pyrR acts by binding to specific sequences on pyr mRNA, causing transcriptional attenuation when intracellular levels of uridine nucleotides are elevated [40].

### Gene regulation

**Figure 8:**
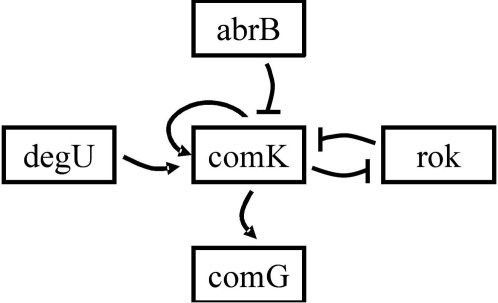
A representation of the known regulation relations in our regulation gene network [41]–[43]. Arrows and blunt arrows represent positive and negative regulation, respectively. At the center of the chart lies the auto-regulatory gene comK. abrB and rok are repressors of comK, and degU is a positive regulator. comK is a positive regulator of the comG operon (comprised of the genes comGA to comGG and yqze) and a repressor of the rok gene.

Regulatory mechanisms controlling expression of genes are diverse, ranging from the basic unit of regulation, the operon, to activators, repressors, etc. In this section we demonstrate the ability of the FH method to capture regulatory mechanisms within operons, expanding our discussion to inter-operon relations existing mainly due to regulation. We focus on the example of negative regulation by repressors (inhibitors of transcription). We expect to detect strong negative correlations (or anti-correlations) between a repressor gene and the genes that are part of the operon it inhibits. To examine these assumptions, we chose to study the regulation network related to sporulation that has gained much interest over the years [41], [42]. We note that despite the wide accumulated knowledge and the recent progress in understanding this system, the dynamics (function) of the competence network is still not completely understood, especially with regards to its relations with the sporulation network [42], [43]. At the core of the bacterial competence system is comK, a positive auto-regulatory gene occupying a central position in the signal-transduction network of the competence system. Several key components of this network and their known regulatory roles are represented in Figure 8 and described in Table 2.

**Table 2:** Selected genes participating in the competence system [42]–[43].

#	Gene	Operon	Relations in the system	Function
1	comK	comK	Self activator and positive regulator of comG	competence transcription factor (CTF)
2	comGA	comG	Positively regulated by comK	DNA transport machinery
3	comGB			Exogenous DNA binding
4	comGC			DNA transport machinery
5	comGD			
6	comGE			
7	comGF			
8	comGG			
9	yqze			
10	degU	degSU	Positive regulator of comK	two-component response regulator
11	rok	rok	Negative regulator of comK and negatively regulated by comK	repressor of comK
12	abrB	abrB	Negative regulator of comK	transcriptional regulator

At the core of the bacterial competence system is comK, a positive auto-regulatory gene occupying a central position in the signal-transduction network of the competence system with several key components of this network and their known regulatory roles.

To proceed with the investigation of this system, we chose to analyze the relevant part of the expression data: the complete data set consists of response to 37 different antibiotics, divided into functional sub-groups according to their mechanisms of action (stress) on the bacteria [21]. Since we are interested in competence regulation, we decided to analyze gene-expression in response to the sub-group of antibiotics that affect cell division. More specifically, we calculated the inter-gene correlations for the response to the group of antibiotics interfering with DNA topology labeled “topo” [21].

**Figure 9:**
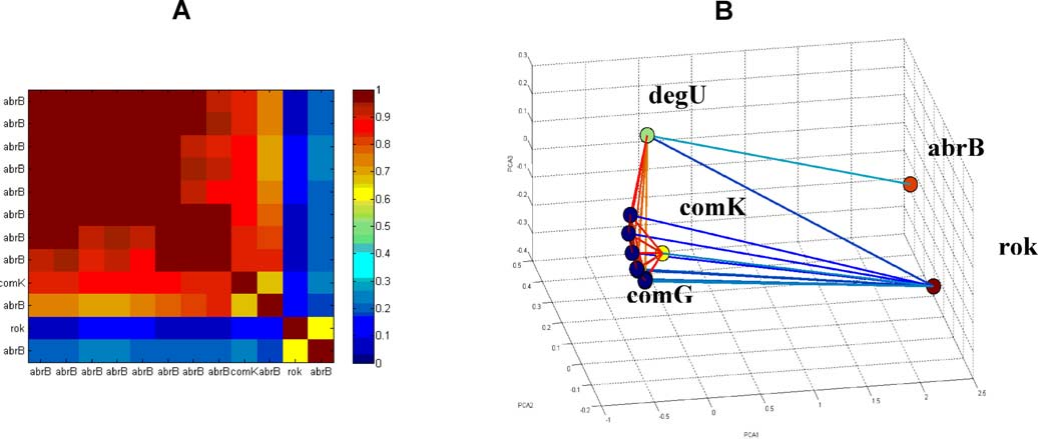
Holographic network of comK regulation. (A) The matrix of normalized correlations for the comK genes and the rok and abrB regulators. (B) The functional relations as revealed in the corresponding 3-D PCA space. The genes of each operon are marked with the same color. Genes with strong positive correlations (correlations above 0.7 in the [0,1] scale), or with strong negative correlations (correlations below 0.3 in the [0,1] scale) are linked. The colors of the lines indicate the level of correlations–blue for negative and red for positive. Note that the abrB and rok genes which are negative regulators of comK are located at a distance from it in the PCA space, and are connected by negative correlations to the comK cluster.

In Figure 9A, 9B we show the resulting matrix of normalized correlations and the corresponding holographic map of gene response in the 3-D PCA space. To single out inhibitory relations we also linked anti-correlated genes–genes that have high negative correlations. To clarify the results, we first present in Figure 9A the matrix of normalized correlations and the holographic network when only two negative regulators–rok, a repressor of comK, and abrB, a negative regulator of both comK and rok.

### Time progress of the gene network response

**Figure 10:**
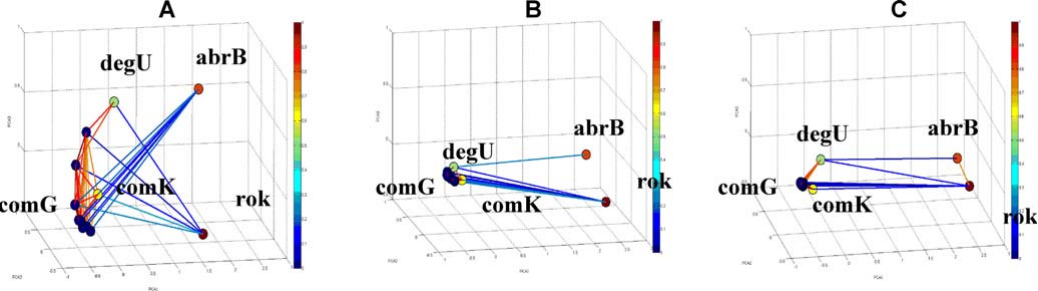
The correlation matrices for the competence gene network for three time stages, projected on the joint FH holographic 3-D of the PCA space for all time points (Figure 9). (A) After 10 minutes of exposure, (B) after 40 minutes of exposure and (C) after 80 minutes of exposure to antibiotics interfering with DNA topology.

In this section we illustrate the ability of the new approach to reveal additional dynamical motifs related to the time progress in the response of the gene network to the antibiotic stress. For this purpose, we examined the response of the competence network, the “topo” antibiotics, at three time points–10, 40 and 80 minutes after exposure. The comparison was performed using the “holographic projection” [33]–[35]. First, we calculate the leading principal components for the joint correlation matrix at all the time points, and then we calculate the correlation matrices for each time point separately. We then project the matrix for each time point onto the 3-dimensional PCA space calculated for the joint correlation matrix. In Figure 10 we show the resulting holographic networks for the three time points after the exposure. It is quite obvious that the holographic network has changed over time. The main change is detected between the 10 and 40 minutes time points. At 80 minutes after exposure, there is an additional effect, as abrB and rok become functionally more similar (closer in the 3-dimension PCA space) and show higher correlations between them.

### Harnessing the method for functional predictions

**Figure 11:**
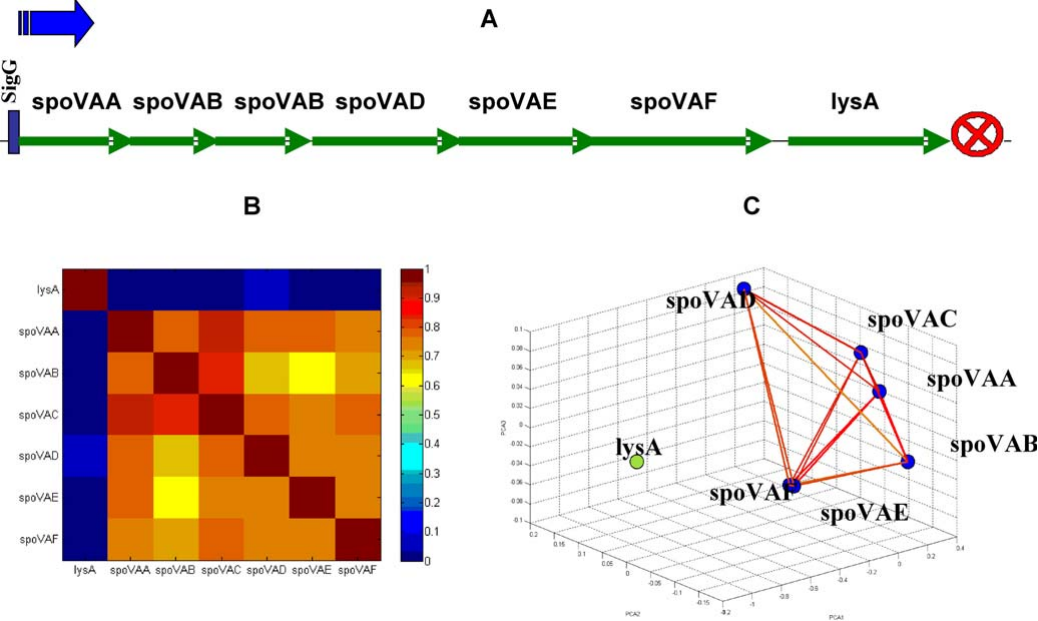
The spoVAA operon [44]. (A) Schematic internal sequential structure presentation. The promoter is represented by a blue arrow, the terminator by a red crossed circle, and the binding site of the activator by a purple rectangle. (B) Matrix of normalized correlations. (C) FH holographic presentation in 3D PCA space, where correlation values above 0.8 are shown in lines.

We demonstrated above the ability of the method to successfully capture known functional relationships between genes belonging to the same operon and also across operons. Based on this demonstrated efficiency of the method, we proceeded to test its ability to predict/reveal unknown functional relations. To test this ability we investigate in this section the spoVAA operon [44]. In Figure 11A we show the currently presumed internal structure of this operon. The matrix of normalized correlations and its holographic networks in the PCA space are shown in Figures 11B and 11C, respectively. Inspecting these results, we observe that lysA has weak correlations with the other genes in the operon. These results are somewhat unexpected since no terminator or regulation factors were found between spoVAF and lysA (Genbank L09228). Azevedo *et al.* found a 2.3 kb transcript originating about 1 kb upstream of the lysA start codon, suggesting that transcription of spoVA continues into the lysA gene. However, the lysA gene is also transcribed monocistronically as a 1.3 kb transcript. A possible explanation might be the existence of a regulation element, a terminator perhaps, between the spoVAF and lysA genes. Another possible explanation can also be the existence of an additional unknown pathway (through another gene) in which the lysA gene acts as a negative regulator of the spoVAA operon. LysA mediates the last step of the lysine biosynthesis [45]. The lysine-mediated gene regulation in bacteria appears to operate via a unique RNA structural element (similar to riboswitch that is involved in the regulation of purin biosynthesis [46]). The LYS element is characterized by its compact secondary structure with a number of conserved helices and extended regions of sequence conservation, which could be necessary for specific metabolite binding [45]. Comparative genomic analysis predicted conserved RNA secondary structures in lysine metabolism genes such as lysC and lysA. Thus, our analysis supports the genomic prediction of a regulatory element adjacent to the lysA gene and transcription of lysA monocistronically.

### Predicting the function of y-genes

A common widespread approach to predicting the function of y-genes (genes whose function is not known) from direct tests, like knockout experiments, is to investigate the homology (genomic sequence and expression profile) between the y-genes and other genes whose function is known. We propose that our analysis method provides a new functional prediction tool for y-genes, by looking at the homology between the functional structures in the PCA space. More specifically, we search for homology between the structures of the functional correlations of two genes in the PCA space with another set of genes (e.g operon). In other words, we look at the homology between the structures of the holographic networks that the two genes form–searching for holographic homology. We note that this holographic homology can be used as a contemporary annotation tool to further enhance the biological knowledge of the gene network.

**Figure 12:**
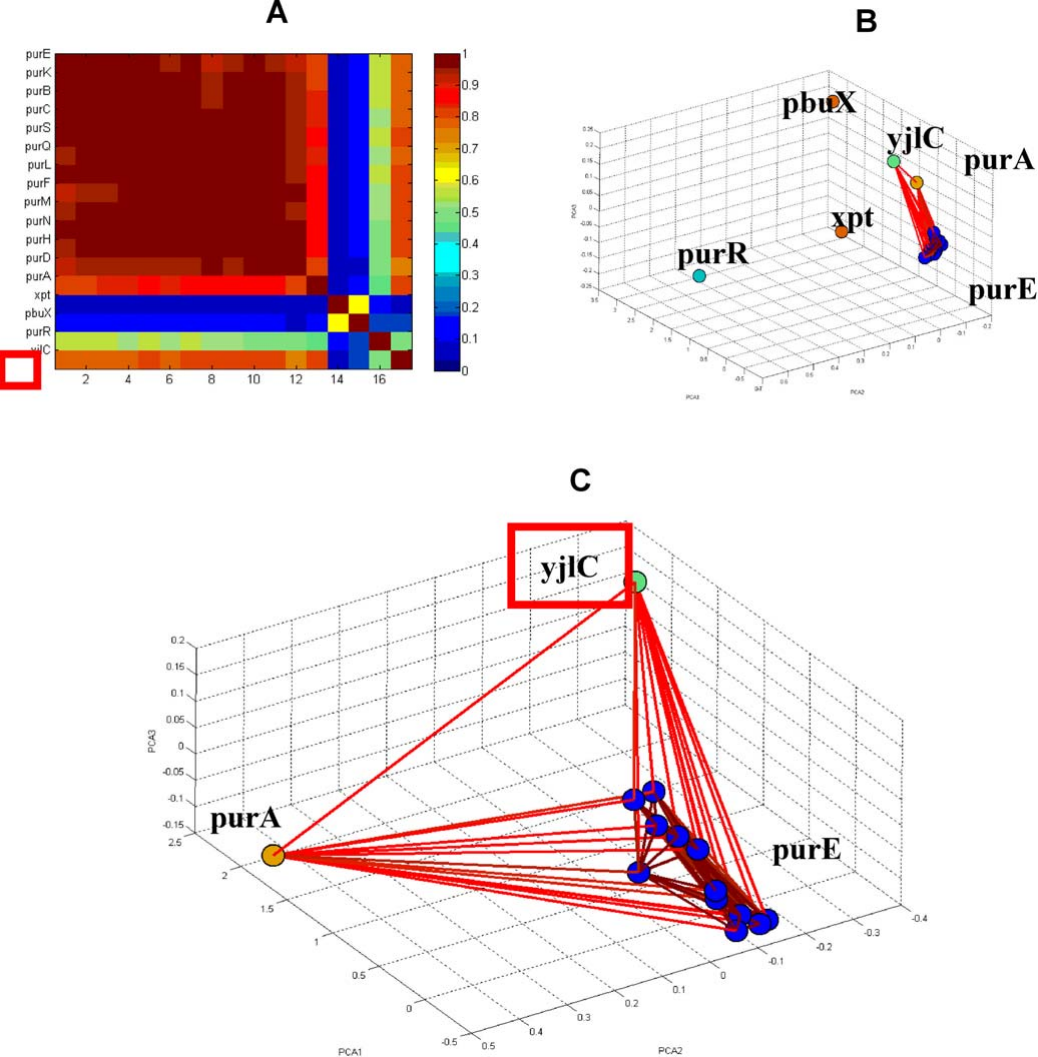
Regulation of purE operon [29]. (A) Matrix of normalized correlations demonstrating the regulation of the xpt, purE and purA operons by purR with low correlation values. (B) FH holographic presentation in PCA space of the genes of purE operon, purA, xpt, pbu, purR and yjlC genes. Note the relationships between the pur operons and the yjlC gene. (C) FH zooming of the purE operon genes and purA and yjlC genes. Note the high resembles between purA and yjlC.

To examine and illustrate this idea, we tested a set of randomly selected y-genes out of the 641 gene that have description unknown [32]. Those genes were added to the operon-clustered genes and co-analyzed. The y-genes that expressed high similarity to known operons, more specifically, y-genes that were clustered with a known operon, were selected and further investigated. Doing so revealed several y-genes that have relatively high correlations to one of the operons. We show here one example for the unknown gene yjlC that the analysis revealed has high correlations with the purE operon and with the known gene purA. The operons in this selected sub-network share the sigA binding factor and the negative regulative factor purR [32]. From the matrix of normalized correlations shown in Figure 12A, it is clear that the unknown gene yjlC (red rectangle) has high correlations with the gene purA and with all the genes of the purE operon, and low correlations with the purR and xpt operons (blue). These selective correlation relationships are further noticeable in the corresponding holographic network shown in Figure 12B, where genes with correlation values above 0.7 are connected by lines. Zooming into the correlation sub-network of the unknown gene yjlC and purA gene and purE operon is shown in Figure 12C.

Our analysis clearly demonstrates that the functional relations of the unknown gene yjlC with the pure operon have high functional similarity (holographic homology) to the functional relations of purA with the purE operon. Such functional homology between yjlC and purA (Figure 12) may indicate that the function of yjlC is related to purine biosynthesis, illustrating the predictive power of the functional holography analysis.

## Discussion

We presented a new, system-level analysis of the complex gene-network response to environmental stress measured by DNA chips. The method is based on the Functional Holography (FH) analysis that was originally developed for analyzing multi-channels recordings of cultured neural networks activity and of recorded brain activity.

We used the method to analyze gene expression of *Bacillus subtilis* exposed to sub-lethal levels of 37 different antibiotics. The matrices of gene correlations were computed and analyzed using the functional holography method. Then, relevant information was extracted from the matrices of normalized correlations by application of the PCA dimension reduction algorithm. The success in retrieving meaningful information proves the assumption that indeed valuable information is embedded in the correlations (similarities) between the expression profiles of different genes.

First we demonstrated the ability of the method to act as an unsupervised and semi-supervised method for identifying the sorting of the genes into operons. We then demonstrated that the approach can also be used to reveal the internal structure of the operons, thus relating the function (expression) to the form. Therefore, it might be possible in the future to use this method as means for unsupervised detection of operons and completion of information about the internal structure of operons for which the internal sequential information is partial. We showed a specific example of using the method to deduce information about the existence of unknown structural motif in the case of the spoVAA operon. Another illustration of the predictive power of the method was in regard to the yjlC unknown gene. We found that the structure of its correlations with the purE operon is very similar to that of the purA operon. These results demonstrated that the method can be used as a prediction tool to reveal functional similarities of unknown genes or operons.

Focusing on specific functional gene network, the competence network, we analyzed the response to a specific class of antibiotic materials–the topo. We found that the dynamical (functional) correlation motifs in the corresponding holographic network (the network in the abstract 3-dimensional space of PCA principal vectors) match the known regulatory relations of the competence system. Performing the analysis at three time points of response, we could identify the progress in the dynamical response. We note that our method, significantly simplifies the interpretation of the complex gene-expression data. Still, this new method requires further development in quantifying the calculated changes.

Here we focused on inspection of the internal structure of operons, but we note that the method can also perform genome-wide analysis by looking at the inter-operon correlations and thus constructing a network of inter-operon functional connectivity.

We also note that the method can be used to inspect clustering for metabolic paths, transcription factors and functional paths. In addition to the analysis that was done for the correlations between genes, one can also investigate the correlations between the effects of the different antibiotics to reveal relations in the effects of different antibiotics or effects of unknown new chemical agents.

## Supporting Information

Appendix S1 (0.72 MB DOC)

## References

[1] Golub TR SD, Tamayo P, Huard C, Gaasenbeek M, Mesirov JP (1999). Molecular classification of cancer: class discovery and class prediction by gene expression monitoring.

[2] Quinlan J (1992). Programs for Machine Learning..

[3] Rumelhart DEHG, Williams RJ, Rumelhart DE, McClelland JL (1986). Learning internal representations by error propagation:. Parallel Distributed Processing, volume 1, pages 318-362.

[4] Furey TS, Cristianini N, Duffy N, Bednarski DW, Schummer M (2000). Support vector machine classification and validation of cancer tissue samples using microarray expression data.. Bioinformatics.

[5] Hartigan J (1975). Clustering Algorithms:.

[6] Everitt BS (1993). Cluster Analysis.

[7] Mirkin B (1996). Mathematical classification and clustering..

[8] Hansen P, Jaumard B (1997). Cluster analysis and mathematical programming.. Math Program.

[9] Pilpel Y, Sudarsanam P, Church GM (2001). Identifying regulatory networks by combinatorial analysis of promoter elements.. Nat Genet.

[10] Getz G, Levine E, Domany E (2000). Coupled two-way clustering analysis of gene microarray data.. Proc Natl Acad Sci U S A.

[11] Eisen M, Spellman P, Brown P, Botstein D (1998). Cluster analysis and display of genome-wide expression patterns. .. PNAS.

[12] Tamayo P, Slonim D, Mesirov J, Zhu Q, Kitareewan S (1999). Interpreting patterns of gene expression with self-organizing maps. .. PNAS.

[13] Herwig R, Poustka AJ, Müller C, Bull C, Lehrach H (1999). Large-Scale Clustering of cDNA-Fingerprinting Data Genome Res.

[14] Alon U, Barkai N, Notterman DA, Gish K, Ybarra S (1999). Broad patterns of gene expression revealed by clustering analysis of tumor and normal colon tissues probed by oligonucleotide arrays.. Proc. Natl. Acad. Sci. USA..

[15] Hartuv E, Shamir R (2000). A clustering algorithm based on graph connectivity.. Inf Process Lett.

[16] Ben-Dor A, Shamir R, and, Yakhini Z (1999). Clustering gene expression patterns.. J Comput Biol.

[17] Sharan R, Maron-Katz A, Shamir R (2003). CLICK and EXPANDER: a system for clustering and visualizing gene expression data Bioinformatics.

[18] Yosef N, Yakhini Z, Tsalenko A, Kristensen V, Borresen-Dale AL (2007). A supervised approach for identifying discriminating genotype patterns and its application to breast cancer data.. Bioinformatics.

[19] Priness I, Maimon O, Ben-Gal I (2007). Evaluation of gene-expression clustering via mutual information distance measure.. BMC Bioinformatics.

[20] Rokhlenko O, Shlomi T, Sharan R, Ruppin E, Pinter RY (2007). Constraint-based functional similarity of metabolic genes: going beyond network topology.. Bioinformatics.

[21] Hutter B, Schaab C, Albrecht S, Borgmann M, Brunner NA (2004). Prediction of Mechanisms of Action of Antibacterial Compounds by Gene Expression Profiling Antimicrobial Agents and Chemotherapy.

[22] Otnes RK, Enochson L (1978). Applied Time Series Analysis..

[23] Jolliffe IT (1986). Principal Component Analysis..

[24] Last M, Szcepaniak PS, Volkovich Z, Kandel A (2006). In Advances in Web Intelligence and Data Mining.. Springer Studies in Computational Intelligence.

[25] Antelmann H, Scharf C, Hecker M (2000). Phosphate Starvation-Inducible Proteins of Bacillus subtilis: Proteomics and Transcriptional Analysis J Bacteriol.

[26] Fawcett P, Eichenberger P, Losick R, Youngman P (2000). The transcriptional profile of early to middle sporulation in Bacillus subtilis Microbiology.

[27] Ye RW, Tao W, Bedzyk L, Young T, Chen M (2000). Global Gene Expression Profiles of Bacillus subtilis Grown under Anaerobic Conditions.. J BACTERIOL.

[28] Helmann JD, Winston Wu MF, Kobel PA, Gamo FJ, Wilson M (2001). Global Transcriptional Response of Bacillus subtilis to Heat Shock JOURNAL OF BACTERIOLOGY,.

[29] Lee J, Zhang S, Saha S, Anna SS, Jiang C (2001). RNA Expression Analysis Using an Antisense Bacillus subtilis Genome Array J Bacteriol.

[30] Ogura M, Yamaguchi H, Yoshida K, Fujita Y, Tanaka T (2001). DNA microarray analysis of Bacillus subtilis DegU, ComA and PhoP regulons: an approach to comprehensive analysis of B. subtilis two-component regulatory systems Nucleic Acids Res.

[31] Salgado H, Gama-Castro S, Peralta-Gil M, Diaz-Peredo E, Sanchez-Solano F (2006). RegulonDB (version 5.0): Escherichia coli K-12 transcriptional regulatory network, operon organization, and growth conditions.. Nucleic Acids Res.

[32] Sierro N, Makita Y, De Hoon M, Nakai K (2007). DBTBS: a database of transcriptional regulation in Bacillus subtilis containing upstream intergenic conservation information.. Nucleic Acids Research Advance Access published online on October.

[33] Baruchi I, Grossman D, Volman V, Hunter J, Towle VL (2006). Functional holography analysis: Simplifying the complexity of dynamical networks CHAOS.

[34] Baruchi I, Ben-Jacob E (2004). Functional holography of recorded neuronal networks activity.. Neuroinformatics.

[35] Baruchi I, Towle VL, Ben-Jacob E (2004). Functional holography of Complex Networks Activity—From Cultures to the Human Brain.. Complexity.

[36] Dubes R, Jain A (1980). “Clustering methodologies in exploratory data analysis” Advances in Computers.

[37] Gidskehaug L, Anderssen E, Flatberg A, Alsberg BK (2007). A framework for significance analysis of gene expression data using dimension reduction methods.. BMC Bioinformatics.

[38] Boulesteix AL, Strimmer K (2007). Partial least squares: a versatile tool for the analysis of high-dimensional genomic data.. Brief Bioinform.

[39] Chen NY, Jiang SQ, Klein DA, Paulus H (1993). Organization and nucleotide sequence of the Bacillus subtilis diaminopimelate operon, a cluster of genes encoding the first three enzymes of diaminopimelate synthesis and dipicolinate synthase.. J Biol Chem.

[40] Chander P, Halbig KM, Miller JK, Fields CJ, Bonner HKS (2005). Structure of the Nucleotide Complex of PyrR, the pyr Attenuation Protein from Bacillus caldolyticus, Suggests Dual Regulation by Pyrimidine and Purine Nucleotides J Bacteriol.

[41] Stragier P, Losick R (1996). Molecular genetics of sporulation in Bacillus subtilis.. Annu Rev Genet.

[42] Errington J (1993). Bacillus subtilis sporulation: regulation of gene expression and control of morphogenesis.. Microbiol Rev.

[43] Grossman AD (1995). Genetic Networks Controlling the Initiation of Sporulation and the Development of Genetic Competence in Bacillus Subtilis.. Annu Rev Genetics.

[44] Azevedo V, Sorokin A, Ehrlich SD, Serror P (1993). The transcriptional organization of the Bacillus subtilis 168 chromosome region between the spoVAF and serA genetic loci.. Mol Microbiol.

[45] Rodionov DA, Vitreschak AG, Mironov AA, Gelfand MS (2003). Regulation of lysine biosynthesis and transport genes in bacteria: yet another RNA riboswitch?. Nucleic Acids Res.

[46] Mandal M, Boese B, Barrick JE, Winkler WC, Breaker RR (2003). Riboswitches control fundamental biochemical pathways in Bacillus subtilis and other bacteria.. Cell.

[47] Golub G, Kahan W (1965). Calculating the Singular Values and Pseudo-Inverse of a Matrix.. Journal of the Society for Industrial and Applied Mathematics: Series B, Numerical Analysis.

