# Supplementary material for: Genome Holography: Deciphering Function-Form Motifs from Gene Expression Data
Source: PLoS One. 2008 Jul 16;3(7):e2708. doi: 10.1371/journal.pone.0002708 (PMC2444029; doi:10.1371/journal.pone.0002708)
Supplement: Appendix S1 — (0.72 MB DOC) [file pone.0002708.s001.doc]

Appendix S1: More details about the analysis methods

**The dendrogram clustering algorithm:** As is mentioned in the text, the dendrogram clustering algorithm was used to identify sub-groups of highly correlated genes. This method described is based on the correlation distances D(i,j) between genes (*i*) and (*j*) – the Euclidian distance between the correlations of genes (i) and (j) with all other genes defined by:

D(i,j) = {N^-1^∑[C(i,l)-C(j,l)]^2^}^1/2^  (A-1)

Note that the correlation distance between two components depends both on the correlation in the response of the two genes as well as on the relative correlations between the responses of each one of the genes with the other genes. Therefore the correlation distances between two components can provide additional important information beyond their direct correlation.

After calculating the distance matrix, a hierarchical tree is constructed [11]. This dendrogram links genes by distance hierarchy. The pair of genes with the shortest distance is linked, followed by the second pair, etc [11]. Then the genes are reordered such that genes with shorter correlation distance are located closer. Consequently the correlation matrix is sorted (reordered) according to the correlation distances between the genes. Sub-groups of highly correlated genes correspond to distinct clusters in the sorted correlation matrix. In the calculations presented here we used the MATLAB algorithm that is based on the nearest neighbor scheme for reordering the genes. The results of the algorithm are a dendrogram plot (Figure 2B, Figure 3B). The dendrogram clustering procedure is very illuminative in rearranging the data into sub-groups of similar characteristics. This enables the focusing on subsets of the original data containing well defined sub-groups of genes.

**Dimension reduction:** One of the problems with high-dimensional datasets is that, in many cases, not all the measured variables are “important” for understanding the underlying phenomena of interest. Using dimension reduction, it is possible to capture the essential information and represent the data visually in three dimensions [23], [37], [38]. Usually the dimension reduction is done by first identifying the principal directions in the correlation space that represents the directions of maximal variations. The next step is to project the nodes on a few (typically 1-3) leading directions. Here we used the Principal Component Analysis (PCA) algorithm [23] that is widely used for visualizing information of high dimensionality. The algorithm approximates the "best" linear representation of the information in lower dimensionality.

It can be shown [35] that the analytic solution of variance maximization involves the digitalization of the correlation matrix. The eigen-vector with the highest associated eigen-value represents the direction in which the most variance is captured. The following eigen-vector with the second highest eigen-value represents the next direction, etc.

The PCA procedure involves the following steps: The correlation matrix is diagnolized and ordered in descending eigen-values:

$C=U\cdot G\cdot U^{T}$ $G_{ii}>G_{jj}$ for any i>j (A-2)

The algorithm used for diagonalization is the Singular Value Decomposition (SVD) algorithm [47]. Once the eigen-vectors are calculated, the original data is projected on these vectors:

$C_{PCA}=U^{T}\cdot C$ (A-3)

This new C_PCA_ matrix is a description of the original C matrix using not the original axis (each corresponds to the correlations of a specific component with all other ones), but a superposition of these axes (the eigen-vectors). The first few eigen-vectors that represent the majority of the variation are called the Primary Vectors (PV's). It is then possible to plot the new C_PCA_ matrix using only the first PV's.

Appendix S2: Comparison between analysis of the correlation matrices and the expression matrices

The analysis approach includes two main elements: 1. Analysis of the gene correlation matrices instead of the response matrices (the asymmetric matrices in which the row corresponds to the genes and the column to the different antibiotic treatments). 2. The retrieval of information (lost in the information reduction algorithm) by linking the nodes (genes) with lines that represent the gene correlations.

To signify the advantage of the above elements we present here comparison with similar analysis (clustering and projection on a reduced 3-D space) of the gene expression matrices. We note that since these are non symmetric matrices we used the SVD algorithm instead of the PCA. We show results both for inter and intra operon relations. We show that the method main advantage is in revealing functional relations between genes within the operons.

First we study the expression matrix of 66 genes, 59 that belong to well defined operons (same as in Figure 5) and additional 7 randomly chosen unknown genes (in bright orange color). The results of the SVD analysis for gene expression matrix are shown in Figure A-1A and the results for the FH analysis of the corresponding correlation matrix are shown in Figure A-1B. The importance of inclusion of the correlations also in the case of the SVD analysis is illustrated in Figure A-1C. We notice that also for this case connecting the genes according to the correlations retains much of the information which is lost in the dimensional reduction process. This key feature helps distinguish between the different clusters. We note that as the percentage of information contained in the three leading principal vectors lessens, the effect of connecting the nodes according to the original correlation values becomes more and more important.


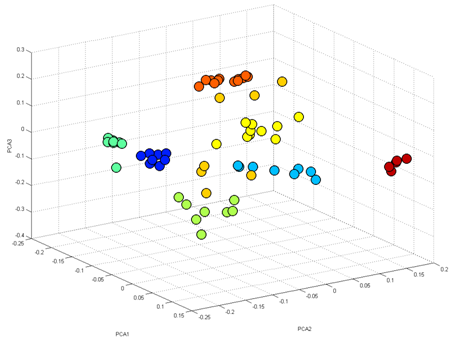

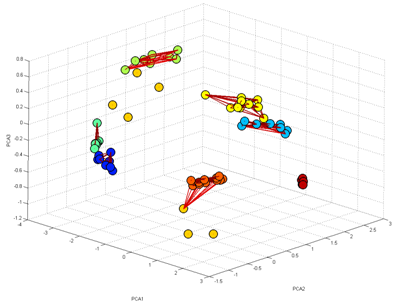

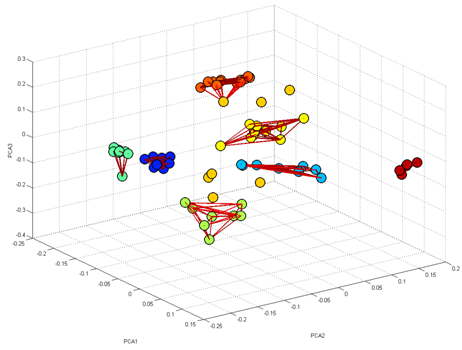


**A**

**B**

**C**

**Figure A-1:** The importance of analyzing the gene correlations. The results are for 66 genes, 59 that belong to well defined operons (same as in Figure 5) and additional 7 that are randomly chosen unknown genes (in bright orange color). In (A) we show dimension reduction using the SVD algorithm on the gene expression matrix. In (B) we show the results of the FH analysis of the corresponding matrix of normalized correlations. In (C) we show the same results as in (A) but when the genes are linked according to the correlations. Note that only by adding the correlation information we deciphered the functional role of one of the unknown gene (linked to the red operon at the bottom in (B) and the top in (C)). We also note that the results are presented in an angle of view that makes the SVD results clearer. At a different angle of view the operons in (B) look further.

For more crucial test we present in Figures A-2 and A-3 comparison of the analysis for the competence pathway. In Figure A-2 we show the results when taking the gene expression matrix for response to the topo antibiotics as was studied in the article.

**A**

**B**

**C**

**Figure A-2:** Comparison for the comK regulation in response to the 'topo' antibiotics. (A) The FH results presented in the article (included here to ease the comparison). The genes of each operon are marked with the same color. Genes with strong positive correlations (correlations above 0.7 in the [0,1] scale), or with strong negative correlations (correlations below 0.3 in the [0,1] scale) are linked. The colors of the lines indicate the level of correlations – blue for negative and red for positive. Note that the abrB and rok genes which are negative regulators of comK are located at a distance from it in the PCA space, and are connected by negative correlations to the comK cluster. (B) Applying the SVD algorithm to the gene expression matrix. (C) The same as (B) but linking the genes according to the correlations.

For further comparison we show in Figure A-3 results for the competence pathway when taking the response to all antibiotics. The advantage of considering the correlations is most transparent in this example.


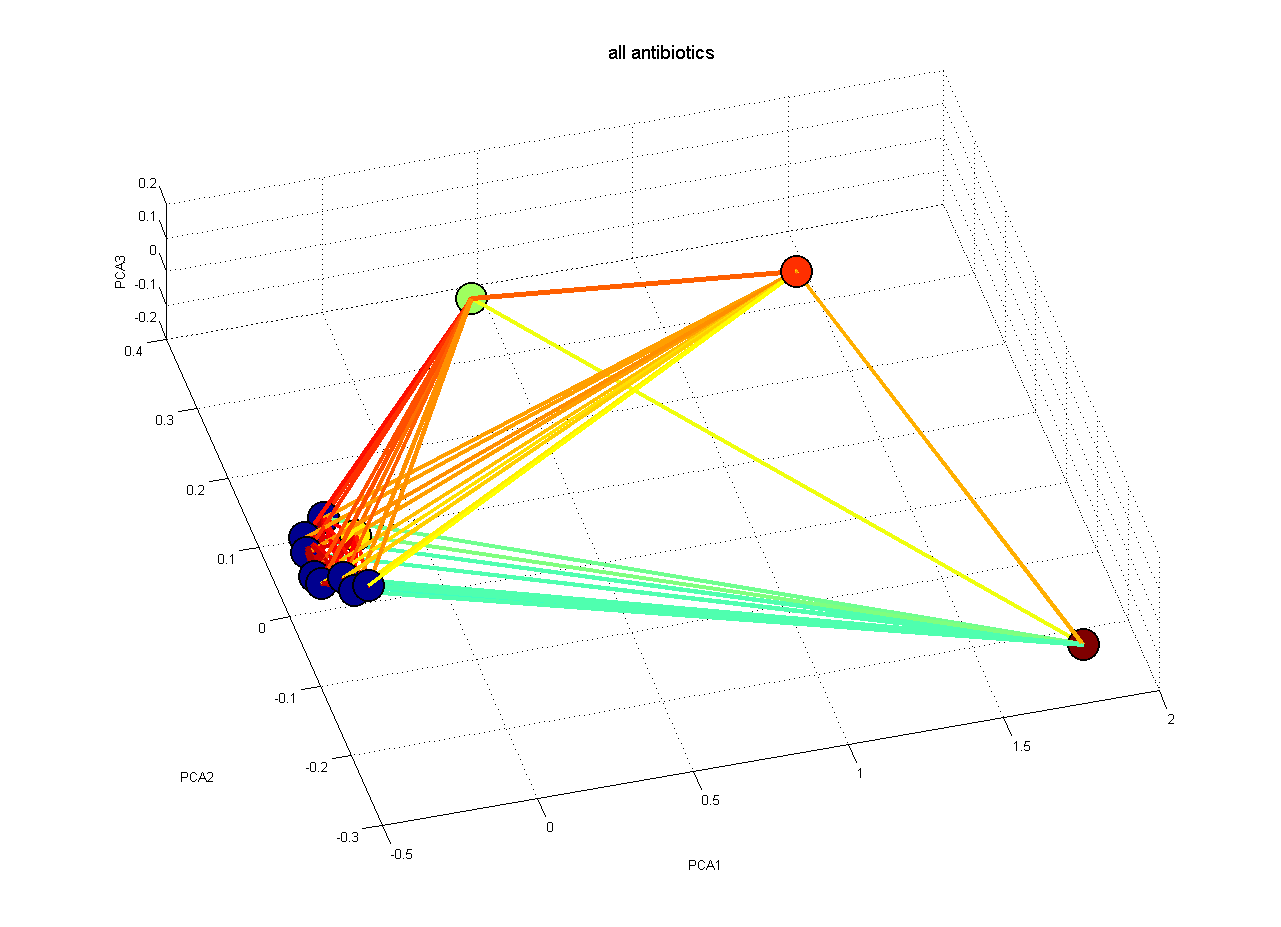


**comG**

**comK**

**degU**

**rok**

**abrB**


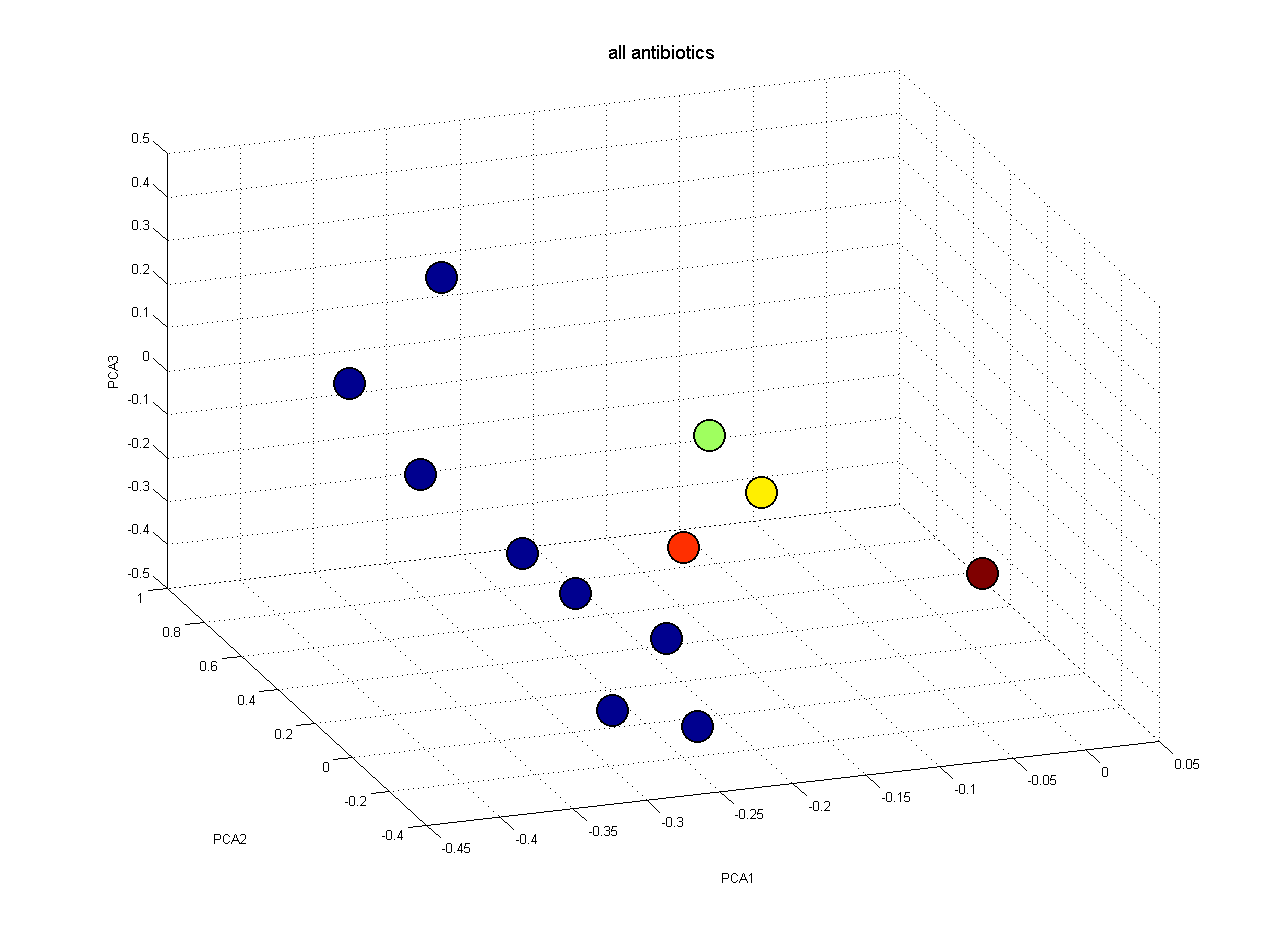


**comG**

**comK**

**rok**

**abrB**

**degU**


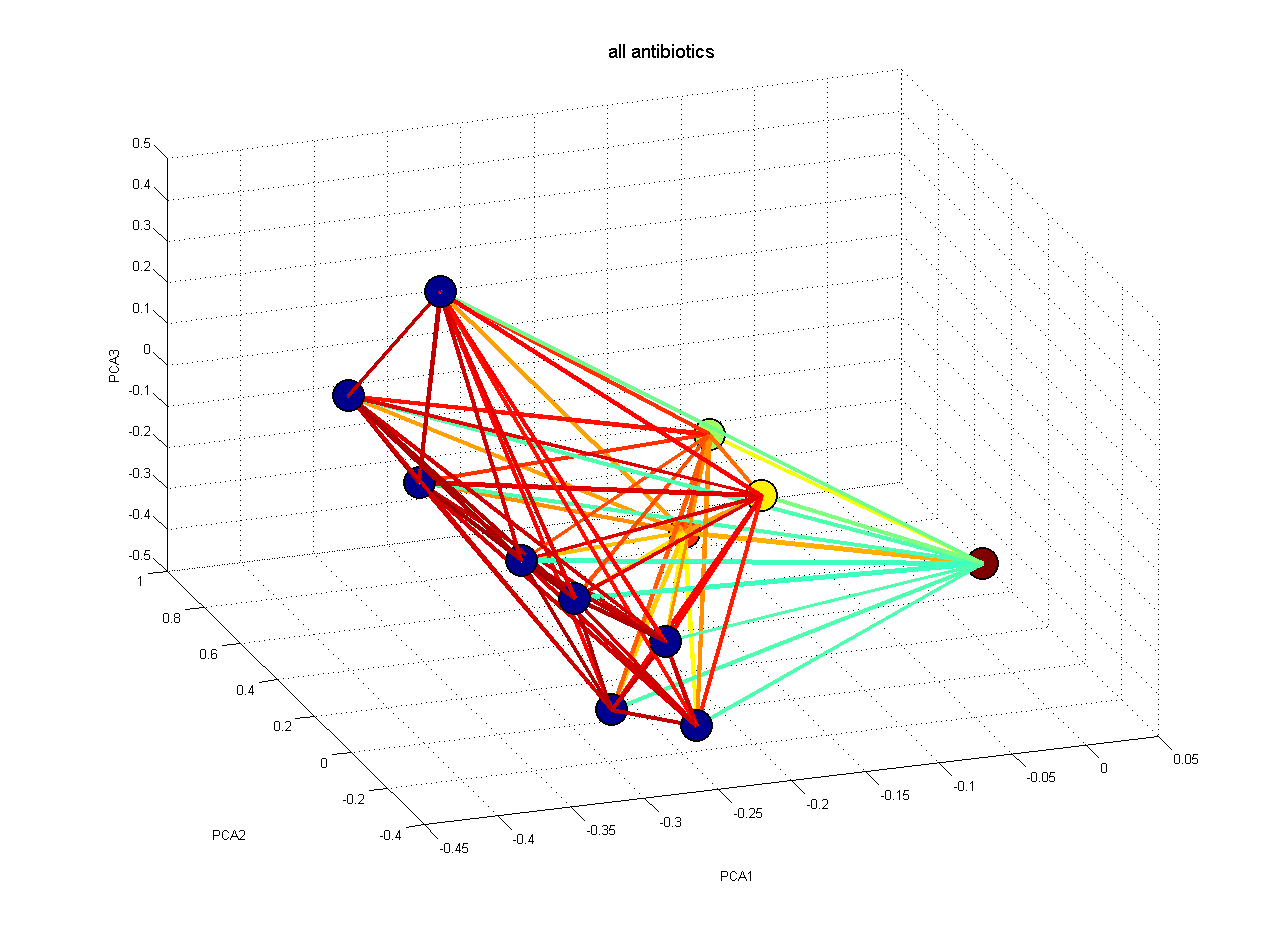


**comG**

**comK**

**degU**

**rok**

**abrB**

**A**

**B**

**C**

**D**


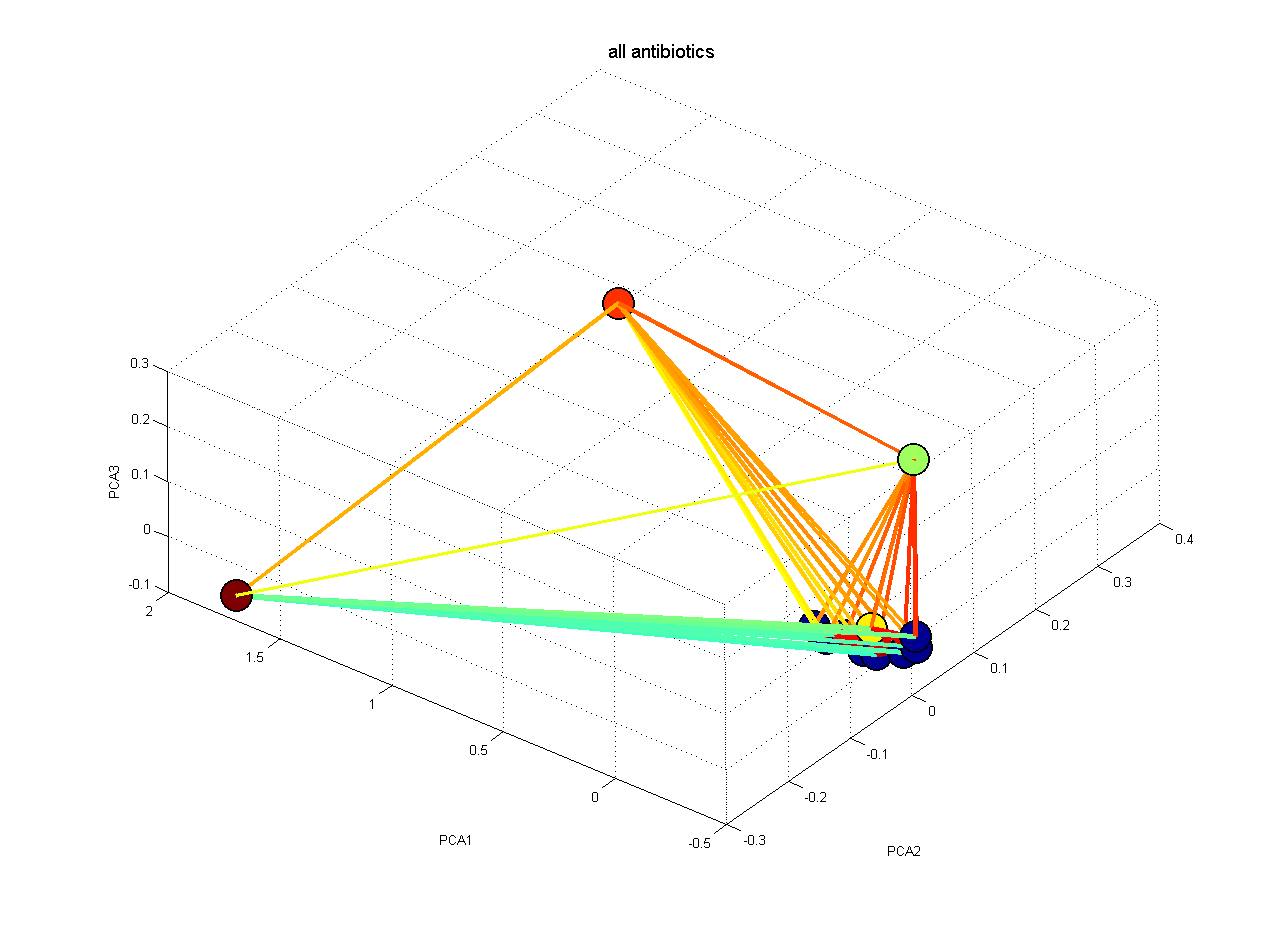


**comG**

**comK**

**degU**

**rok**

**abrB**

**Figure A-3:** Holographic network of comK regulation in response to all antibiotics. (A) Results of the SVD analysis of the gene expression matrix. (B) The same as (A) but when the genes are linked according to the correlations. (C) Results of the FH analysis of the corresponding matrix of normalized correlations. (D) The same as (C) but from a different view angle to make the functional (regulation) motifs more transparent. The genes of each operon are marked with the same color. Genes with strong positive correlations (correlations above 0.7 in the [0,1] scale), or with strong negative correlations (correlations below 0.3 in the [0,1] scale) are linked. The colors of the lines indicate the level of correlations – blue for negative and red for positive. Note that the abrB and rok genes which are negative regulators of comK are located at a distance from it in the PCA space, and are connected by negative correlations to the comK cluster.
